# Unleashing Nature’s potential: a computational approach to discovering novel VEGFR-2 inhibitors from African natural compound using virtual screening, ADMET analysis, molecular dynamics, and MMPBSA calculations

**DOI:** 10.3389/fmolb.2023.1227643

**Published:** 2023-09-20

**Authors:** Soukayna Baammi, Achraf El Allali, Rachid Daoud

**Affiliations:** ^1^ African Genome Center, Mohammed VI Polytechnic University, Ben Guerir, Morocco; ^2^ Chemical and Biochemical Sciences-Green Processing Engineering, Mohammed VI Polytechnic University, Ben Guerir, Morocco

**Keywords:** VEGF receptor, virtual screening, molecular simulations, ADMET analysis, MMPBSA

## Abstract

One of the characteristic features of cancer is angiogenesis, the process by which new, aberrant blood vessels are formed from pre-existing blood vessels. The process of angiogenesis begins when VEGF binds to its receptor, the VEGF receptor (VEGFR). The formation of new blood vessels provides nutrients that can promote the growth of cancer cells. When it comes to new blood vessel formation, VEGFR2 is a critical player. Therefore, inhibiting VEGFR2 is an effective way to target angiogenesis in cancer treatment. The aim of our research was to find new VEGFR-2 inhibitors by performing a virtual screening of 13313 from African natural compounds using different *in silico* techniques. Using molecular docking calculations and ADMET properties, we identified four compounds that exhibited a binding affinity ranging from −11.0 kcal/mol to −11.5 Kcal/mol when bound to VEGFR-2. These four compounds were further analyzed with 100 ns simulations to determine their stability and binding energy using the MM-PBSA method. After comparing the compounds with Regorafenib, a drug approved for anti-angiogenesis treatment, it was found that all the candidates (EANPDB 252, NANPDB 4577, and NANPDB 4580), with the exception of EANPDB 76, could target VEGFR-2 similarly effectively to Regorafenib. Therefore, we recommend three of these agents for anti-angiogenesis treatment because they are likely to deactivate VEGFR-2 and thus inhibit angiogenesis. However, it should be noted that the safety and suitability of these agents for clinical use needs further investigation, as the computer-assisted study did not include *in vitro* or *in vivo* experiments.

## Introduction

Angiogenesis is a critical pathogenic process in many disease conditions (Niu and Chen, 2010). For instance, in cancer, angiogenesis is essential for the development and growth of the cancer cells (Nieves et al., 2009). Normally, the cancer cells relied on the formation of new blood vessels as the source of oxygen and nutrients for the developing tumor (Nishida et al., 2006). The process of angiogenesis begins when Vascular Endothelial Growth Factor (VEGF) binds to its receptors particularly the VEGFR-2 receptor (Nieves et al., 2009). Specifically, the overexpression of VEGF is associated with autophosphorylation of VEGFR-2 receptor in malignancy (Nieves et al., 2009). The VEGF and other growth factors produced by the tumor results in the production of new blood vessels which allows the cells to grow exponentially (Rajabi and Mousa, 2017). Under the influence of VEGF, the vasculature formed are abnormal resulting into abnormal conditions. Hence, the role of this growth factor in vasculature makes it a target for cancer treatment (Carmeliet, 2005).

The main goal of inhibiting tumor angiogenesis is to deprive cancer cells of the nutrients and oxygen they need to grow (Rajabi and Mousa, 2017). The therapeutic value of VEGFR-targeted cancer therapy is described by a large body of clinical evidence. When the VEGFR-2 pathway is blocked, it has a significant anti-angiogenic effect on human cancer (Ansari et al., 2022). Currently, drugs designed to block VEGF and its receptor are approved for the treatment of cancer and ocular diseases. These drugs include anti-VEGF antibodies such as bevacizumab, ranibizumab, and pegaptanib, and VEGFR inhibitors such as sunitinib, sorafenib, Regorafenib, and pazopanib (Carmeliet and Jain, 2011; Yoo and Kwon, 2013; Schmieder et al., 2014). Despite their efficacy, these drugs have limited efficacy and may develop resistance over time. For example, bevacizumab is known to cause severe inflammation in the eye (Sharma et al., 2012), while sunitinib can cause various side effects, such as thrombocytopenia and hypertension, in the treatment of metastatic renal cell carcinoma (Motzer et al., 2006).

Traditional medicine has been used for thousands of years, with a close relationship between medicinal and natural products. Extracts from different parts of medicinal plants were traditionally used in the treatment of different ailments (Rizvi et al., 2022). The awareness of the healing capacity of medicinal plants led to the discovery of phytochemicals (Thakur et al., 2020). The advent of technology led to the isolation of these chemicals that were believed to play a role in the plant healing process (Sasidharan et al., 2011). These natural compounds were reported to have several biological activities and serve as leads for the discovery of new drug candidates. For instance, some of the compounds were reported to exhibit anticancer (Prakash et al., 2013), antidiabetic (Ivorra et al., 1989; Li et al., 2004), antioxidant (Lourenço et al., 2019), anti-inflammatory (Ola et al., 2020), antitumor activities among others. Hence, natural products serve as the richest source of new classes of molecules for biological research. These molecules will continue to play a pivotal role in ethnopharmacology (Beutler, 2009). It is believed that many of these compounds are yet to be discovered. Also, the biological and pharmacological activities of the known compounds are not fully explored (Dias et al., 2012).

Although biochemical and cellular assays can evaluate more synthetic and natural compounds, their empirical screening is limited (McInnes, 2007), making computational methods important in drug discovery. Virtual screening allows searching for hits from chemical collections acquired for biological activity assays. By filtering vast virtual libraries, virtual screening based on molecular docking with a target protein of empirically known structure is becoming a standard approach for identifying promising lead molecules in drug discovery projects (Jain, 2004; Shoichet, 2004). To better understand the physical basis of interactions between protein receptors and their small molecule inhibitors, molecular dynamics simulation has become a fundamental method in drug discovery. The stability of receptor-ligand complexes can be evaluated by molecular dynamics simulations. In addition, this simulation considers the role played by individual amino acid residues.

**FIGURE 1:**
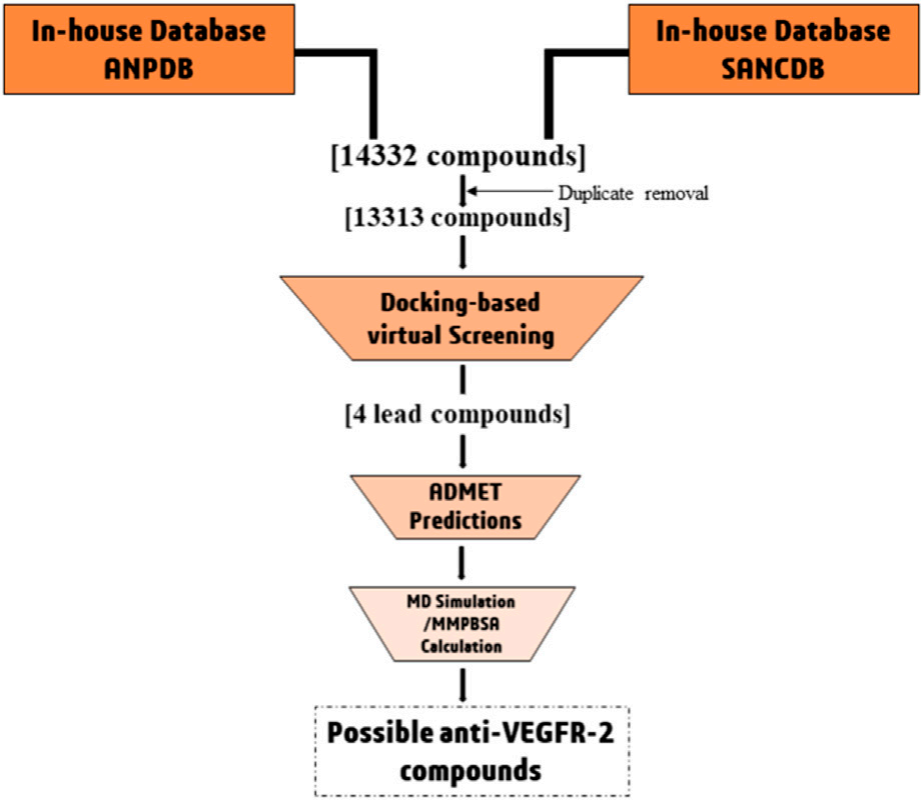
Workflow illustrating the screening process used to identify novel VEGFR-2 inhibitors.

Due to the limitations of currently available drugs, the development of small molecule inhibitors that can block VEGFR signaling is an attractive strategy to prevent angiogenesis. This approach is less likely to cause long-term toxicity (Byrne et al., 2005). In this study, we aim to identify potential VEGFR-2 inhibitors from natural sources using computational methods. This could help in finding alternative drug candidates that could be effective against the process of angiogenesis. The approach employed includes the collection of plant-based naturally occurring African compounds, followed by virtual screening of compounds using molecular docking approach against VEGFR-2 receptor to identify potential lead compounds. Finally, the lead compounds were subjected to molecular dynamics simulations and MMPBSA calculations to identify their stability Figure 1.

## Material and methods

### Database preparation

We performed virtual screenings with compounds contained in the African Natural Products Database (ANPDB) (http://african-compounds.org/nanpdb/) and South African Natural Compounds Database (SANCDB) (https://sancdb.rubi.ru.ac.za/). It is important to note that the ANPDB contains information on compounds from different parts of Africa (Ntie-Kang et al., 2017; Simoben et al., 2020), while the SANCDB is specifically for natural compounds collected from South African (Beutler, 2009; Ola et al., 2020). Altogether, a total of 14332 natural compounds were retrieved from the databases. After removal of duplicate, the compounds reduced to 13313 (Ntie-Kang et al., 2017; Simoben et al., 2020). Thereafter, the compounds were downloaded in “Sdf” format protonated and subjected to energy minimization (Aljuaid et al., 2022). Subsequently, the compounds were converted to pdbqt format using the python script (*mk_prepare_ligand.py* found in MGLTools 1.5.6.) in preparation for molecular docking studies.

### VEGFR-2 preparation

The crystal structures of the protein were retrieved from the Protein Data Bank with the respective PDB codes: 4ASD and 4AGC. A multiple sequence alignment demonstrated that both 4AGC and 4ASD exhibit 100% sequence similarity and are well aligned when superimposed. Both structures share conserved residues within their active sites and the docking results of Regorafenib, an FDA-approved drug, with both 4AGC and 4ASD showed similar interactions. Due to the fewer missing residues in 4ASD, we selected it for our subsequent studies. To address the missing residues, we employed “Build Structure” from Chimera’s modeling tools to rectify the protein structure. Prior to the docking analysis, we prepared the VEGFR-2 protein by removing water molecules, ions, and the ligand (Tabti et al., 2023). The protonation of the amino acids was analyzed on the H++ server, and the hydrogen atoms were added to the whole structure.

### Molecular docking

Virtual screening of retrieved compounds against VEGFR-2 was conducted using AutoDock Vina (Abdelkader et al., 2022). Before commencing the docking studies, MGLTools 1.5.6 was used to convert the VEGFR-2 structure from its native format, pdb, to the docking-ready format, pdbqt by adding the polar hydrogen atoms and Gasteiger charges to the protein chain (Baammi et al., 2022). Except for the exhaustiveness parameter, which was set to 100, all default values were retained. The exhaustiveness plays a role in controlling thoroughness of the search space exploration during molecular docking. Although the default value of exhaustiveness is 8, increasing it to 100 led to an increase in the reproducibility of our docking results (Devaurs et al., 2019). The grid box spacing was set to 0.375 Å, the center to (−24.611 Å, −0.388 Å, −10.929 Å), and the lattice size to 20 Å × 20 Å × 20 Å. Nine poses were constructed for each protein-ligand complex based on docking affinity. The discovery Studio Viewer was used to display and analyze the docking results to find the important interactions between the ligands and the protein binding site (Baammi et al., 2023). In addition, the co-crystalline ligand was re-docked as an inhibitor of VEGFR-2 using the above parameters and values following by comparing the RMSD (root-mean-square deviation) of the heavy atoms between the docked pose and the crystallographic pose of the ligand (Xu and Meroueh, 2016). The nine best ranked poses were visualized using Discovery Studio Visualizer version 17.2 and PyMol version 1.1. The best docked complexes with the lowest docking score and the most favorable interactions were used for molecular dynamics simulations (Hosseini and Amanlou, 2020).

### Pharmacokinetic (ADME) and toxicological predictions

The determination of compounds ADMET properties has a crucial impact in drug design and discovery (Guan et al., 2019). Information on these properties helps in understanding the pharmacokinetics and pharmacodynamics of a drug candidate (Walker, 2004). Of course, 60% of lead compounds fail during screening in the drug discovery pipeline due to their unacceptable ADMET properties. Hence, early prediction of these properties would result in substantial cost savings within the field of drug research (Mandlik et al., 2016). Some of the parameters evaluated for the ADMET includes adsorption, distribution, metabolism, excretion, and toxicity (En-nahli et al., 2022). To accurately predict drug efficacy, penetration through the blood-brain barrier, absorption in the human intestine, CNS permeability, inhibition of cytochrome P450 2D6 and 3A4, hepatotoxicity, and AMES toxicity are also considered (Lagorce et al., 2017). For this study, the ADMET parameters for the four lead compounds were predicted using the pkCSM web server (Pires et al., 2015). These compounds were further subjected to MD simulation studies.

### Molecular dynamics simulation

The behavior of the selected ligands was investigated together with VEGFR-2 over a period of 100 ns using the GROMACS software package version 2019.3 (Park et al., 2020) on a high-performance cluster (POWEREDGE C6420, CRC-STACKHPC, XEON PLATNIUM 8276L 28C 2.2GHZ, MELLANOX INFINIBAND HDR100)). The CHARMM27 force field was used for the protein (Lindahl et al., 2010), and the topology for the ligands was generated using the Swissparam server (Zoete et al., 2011). Prior to neutralization in the system with counterions, each complex was resolved in a dodecahedral box (1.0 nm) using the TIP3P water model. The steepest descent method was used to achieve both the minimum energy and maximum force, with Fmax set to 1,000 kJ/mol/nm (Al-Khafaji and Taskin Tok, 2020). To equilibrate the system at 300 K and 1 bar, two 100 ps simulations were performed in rapid succession using canonical NVT and isobaric NPT ensembles. Subsequently, 100 ns molecular dynamics simulations were performed for each molecule. The output trajectories were generated, and the data files were analyzed to better understand the behavior of the protein.

### MM-PBSA binding energy calculation

The Molecular mechanics Poisson–Boltzmann surface area (MM-PBSA) was used to compute the binding free energies of the complexes (Kumari et al., 2014). The calculations were conducted using the g_mmpbsa script tool (reference (Simoben et al., 2020)), which employs an approach based on the average of two energy values: the solvation energy and the potential energy in a vacuum.

$$∆E\;\left(\text{MM}-\text{PBSA}\right)=∆E_{\text{MM}}+∆G_{\text{solvation}.}$$ (1)

In Equation 1, E_MM_ represents the potential energy in a vacuum, while Gsolvation corresponds to the free solvation energy. The molecular mechanical energy (E_MM_) is determined by considering the contributions of the electrostatic component (E_ele_) and the van der Waals interaction (E_vdW_). The solvation energy is calculated using the polar solvation energy (G_pol_) and the non-polar solvation energy (G_nonpol_). The polar solvation energy (G_pol_) is determined using the Poisson-Boltzmann equation (PB), while the non-polar solvation energy (G_nonpol_) is evaluated based on the solvent-accessible surface area (SASA).

## Result and discussion

### Virtual screening and molecular docking

Computer-aided drug discovery (CADD) relies heavily on molecular docking as a fundamental technique. This method uses computer models to evaluate the interaction between a receptor and the multiple compounds in a virtual environment by using computational algorithms that calculate factors such as binding affinity, energy, and molecular interactions (Yu and Mackerell, 2017). This approach allows researchers to select promising lead molecules for further investigation. To discover potential compounds against VEGFR-2, a molecular docking simulation of the binding pocket of VEGFR-2 was performed using 13363 molecules from African plants. Using the docking method, all compounds were predicted, ranked from lowest to highest docking score, and compared to that of the co-crystallized ligand (−11.0 kcal/mol). Of the 13363 compounds with structural differences, only four compounds had a binding affinity ≤ −11.0 Kcal/mol as Regorafenib in terms of binding energy. Further in-depth molecular studies of the interactions of these ligands with VEGFR-2 are shown in Table 1. It should be noted that the best compounds are those with the lowest docking score and the most beneficial interactions that could fit well into the binding site (Hosseini and Amanlou, 2020).

**TABLE 1:** The analyzed binding affinity for the best six African compounds against the VEGFR-2 protein.

Ligands	Binding affinity	Residues inolved in conventional hydrogen bond formation	Number of hydrogen bond formed	Residues involved in hydrophobic interactions	Residues involved in other interactions
NANPDB 4577	−11.5	Glu885, Ser884, Ala881, Lys868, Asp1046	5	Leu840, Leu889	Asp814, Cys817
EANPDB 76	−11.2	Val914, Asp1046	2	Phe918, Ala866, Leu1035, Val899, Leu889, Cys1024, ILe888, Val848, Phe1047, Val916, Leu840	Ly868, Glu885, Cys1045
NANDB 4580	−11	Arg842, Cys919, Asn923, Ile1044, Leu840	4	Arg1051, Ala866, Val848, Val916, Val899, His1026, Val916, Leu889	Cys1045
EANPDB 252	−11.2	Asp1046, His1026, Ala881	3	Val898, Leu1019, Ile1044	Asp1046, Cys817
Regorfenib	−11	Asp1046, Cys919, Lys868	3	Leu889, Val916, Val848, Val899, Ala866, Leu840, Leu1035	Cys1045, Glu885

**FIGURE 2:**
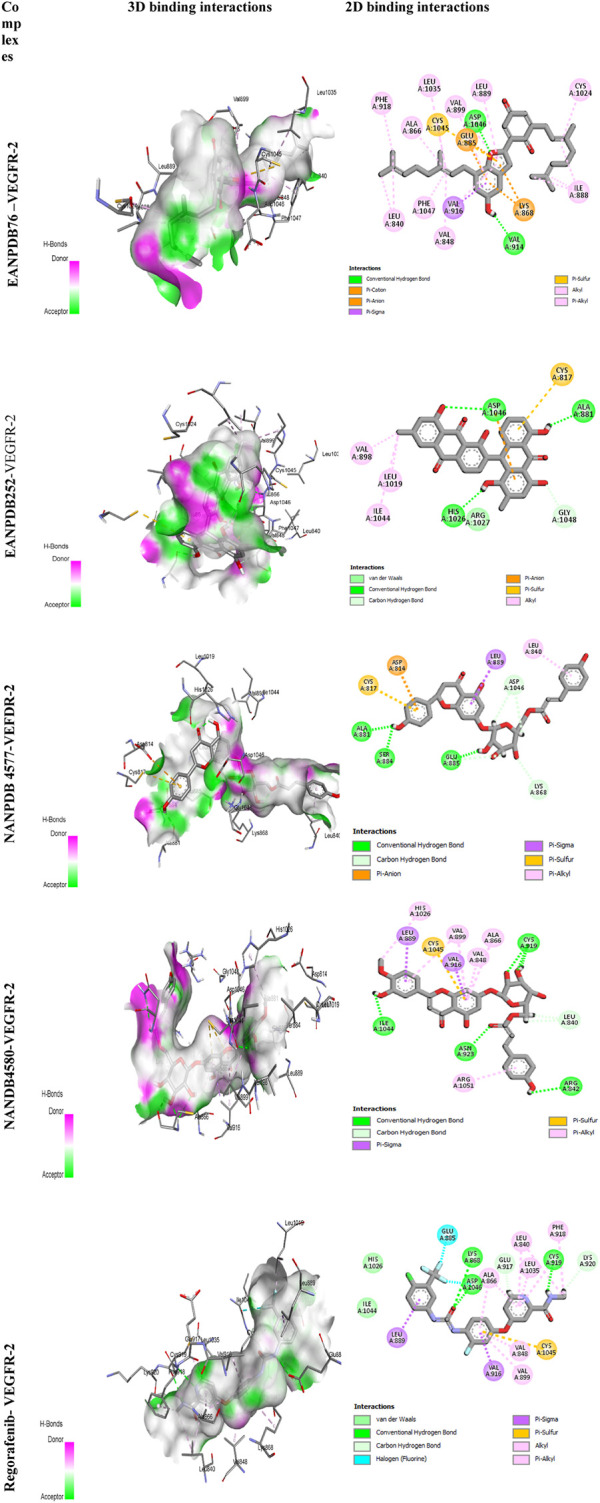
3D and 2D binding interactions of the selected compounds.

The most effective ligand for docking with VEGFR-2 was Naringenin 7-p-coumaroylglucoside (NANPDB 4577) with a binding affinity of −11.5 kcal/mol. There is a strong interaction with VEGFR-2, as evidenced by the presence of four hydrogen bonds between Glu885, Ser884, Ala881, and Lys868, and several hydrophobic, electrostatic, and other interactions between Leu840, Leu889, Asp814, and Cys817. These molecular interactions of NANPDB 4577 with VEGFR-2 are shown in Figure 2. For Naringenin 7-p-coumaroyl glucoside isolated from *Phlomis aurea*, there is no evidence yet of therapeutic use in cancer therapy. Further investigation of the medicinal potential of this compound is recommended in view of our results.

Other compounds, Lettowifuraquinone (EANPDB 76) and Abyquinone B (EANPDB 252), were best docked to VEGFR-2 with a binding affinity of −11.2 kcal/mol. They showed strong interactions with VEGFR-2. EANPDB 76 exhibits two hydrogen bonds with Val914, Asp1046 and several hydrophobic, electrostatic, and other interactions via Phe918, Ala866, Leu1035, Val899, Leu889, Cys1024, Ile888, Val848, Phe1047, Leu840, Ly868, Glu885, and Cys1045. However, EANPDB 252 exhibits three hydrogen bonds with Asp1046, His1026, Ala881, and several hydrophobic, electrostatic, and other interactions via Val898, Leu1019, Ile1044, Gly1048, and Cys817. The molecular interactions of EANPDB 76 and EANPDB 252 with VEGFR-2 are shown in Figure 2. These two compounds, Lettowifuraquinone and Abyquinone B, belong to the class of organic compounds known as quinones, which form an important class of cytotoxins against cancer, tumor, antimicrobials, and antiparasitic effects (Campos-Xolalpa et al., 2021). NANPDB 4580 (chrysoeriol 7-p-coumaroylglucoside) has the same binding affinity as the reference (−11.0 kcal/mol) with four hydrogen bonds with Arg842, Cys919, Asn923, and Ile1044 and eight hydrophobic bonds with Arg1051, Ala866, Val848, Val916, Val899, His1026, Val916, and Leu889, and one electrostatic bond with Cys1045. This molecule belongs to the class of Flavonoids that have been widely used as anticancer (Zhao et al., 2019), antimicrobial, antiviral, antiangiogenic (Camero et al., 2018; Zhao et al., 2018), antimalarial, antioxidants, neuroprotective, antitumor, and anti-proliferative agents (Patel et al., 2018).

### Drug metabolism and toxicity profiling

Poor pharmacokinetic profiles and toxicity concerns are often cited as reasons for excluding lead compounds from preclinical and clinical investigations (Singh, 2006). Therefore, when lead compounds are discovered, it would be advisable to use *in silico* methods to predict the potential toxicity and pharmacokinetic properties of the hit compounds (Salifu et al., 2023). Therefore, the top four compounds from the virtual screening were subjected to evaluation of their absorption, distribution, metabolism, excretion, and toxicity. The ADMET properties of all compounds are listed in Table 2.

**TABLE 2:** Absorption, distribution, metabolism, excretion, and toxicity (ADMET) prediction properties using the pkCSM server.

		Ref	EANPDB 76	EANPDB 252	NANPDB 4577	NANPDB 4580	
Property	Model Name	Predicted Value	Predicted Value	Predicted Value	Predicted Value	Predicted Value	Unit
Absorption	Water solubility	−4.324	−7.538	−2.908	−3.489	−3.16	Numeric (log mol/L)
	Caco2 permeability	0.760	0.633	0.53	0.232	0.915	Numeric (log Papp in 10–6 cm/s)
	Intestinal absorption (human)	88.745	91.755	81.233	53.367	53.576	Numeric (% Absorbed)
	Skin Permeability	−2.73	−2.716	−2.735	−2.735	−2.735	Numeric (log Kp)
	P-glycoprotein substrate	Yes	Yes	Yes	Yes	Yes	Categorical (Yes/No)
	P-glycoprotein I inhibitor	Yes	Yes	Yes	No	No	Categorical (Yes/No)
	P-glycoprotein II inhibitor	Yes	Yes	Yes	Yes	Yes	Categorical (Yes/No)
Distribution	VDss (human)	−0.0131	0.813	−1.148	0.172	−0.364	Numeric (log L/kg)
	Fraction unbound (human)	0	0	0.385	0.096	0.22	Numeric (Fu)
	BBB permeability	−1.676	0.2	−1.187	−1.696	−1.712	Numeric (log BB)
	CNS permeability	−2.064	−1.415	−3.069	−4.081	−4.38	Numeric (log PS)
Metabolism	CYP2D6 substrate	No	No	No	No	No	Categorical (Yes/No)
	CYP3A4 substrate	Yes	Yes	No	No	No	Categorical (Yes/No)
	CYP1A2 inhibitior	No	No	Yes	No	No	Categorical (Yes/No)
	CYP2C19 inhibitior	Yes	Yes	No	No	No	Categorical (Yes/No)
	CYP2C9 inhibitior	Yes	No	No	No	No	Categorical (Yes/No)
	CYP2D6 inhibitior	No	No	No	No	No	Categorical (Yes/No)
	CYP3A4 inhibitior	Yes	Yes	No	No	No	Categorical (Yes/No)
Excretion	Total Clearance	−0.042	0.571	−0.063	0.055	0.258	Numeric (log mL/min/kg)
	Renal OCT2 substrate	No	No	No	No	No	Categorical (Yes/No)
Toxicity	AMES toxicity	No	No	No	No	No	Categorical (Yes/No)
	Max. tolerated dose (human)	0.257	0.335	0.339	0.732	0.672	Numeric (log mg/kg/day)
	hERG I inhibitor	No	No	No	No	No	Categorical (Yes/No)
	hERG II inhibitor	Yes	Yes	Yes	Yes	Yes	Categorical (Yes/No)
	Oral Rat Acute Toxicity (LD50)	2.111	2.526	2.391	2.768	2.809	Numeric (mol/kg)
	Oral Rat Chronic Toxicity (LOAEL)	1.075	2.527	3.228	4.33	4.505	Numeric (log mg/kg_bw/day)
	Hepatotoxicity	Yes	No	No	No	No	Categorical (Yes/No)
	Skin Sensitisation	No	No	No	No	No	Categorical (Yes/No)
	*T.Pyriformis toxicity*	0.301	0.509	0.285	0.285	0.285	Numeric (log ug/L)
	Minnow toxicity	−0.398	−4.181	0.007	1.096	−0.261	Numeric (log mM)

pKCSM is an online tool that we used as a reference for several different variables in our study. According to the data from the pKCSM server, a higher HIA value was obtained for compounds EANPDB 76, EANPDB 252, NANPDB 4577, and NANPDB 4580, suggesting that these compounds may be more absorbed in the intestine after oral administration. To determine whether a compound is mutagenic, the researchers used the AMES toxicity assay. All four compounds tested (EANPDB 76, EANPDB 252, NANPDB 4577, and NANPDB 4580) proved negative in the AMES toxicity test. According to hepatotoxicity prediction research, the new chemical poses no risk to liver function. The group of isoenzymes that includes cytochrome P450 (CYP) is the key factor in pharmacokinetics. It plays an important role in the metabolic processes of numerous substances, such as drugs, bile acids, steroid hormones, fatty acids and carcinogens. Some of the substances tested may be able to prevent certain cytochrome P450 isoforms from functioning properly. These isoforms are important enzymes involved in the metabolism of various drugs and can cause negative interactions and side effects when combined with other drugs. In silico data suggests that all tested compounds (EANPDB 76, EANPDB 252, NANPDB 4577, and NANPDB 4580) are neither substrates nor inhibitors of CYP2D6. However, EANPDB 76 was found to be an inhibitor and substrate of CYP3A4.

### Molecular dynamics simulation analysis

**FIGURE 3:**
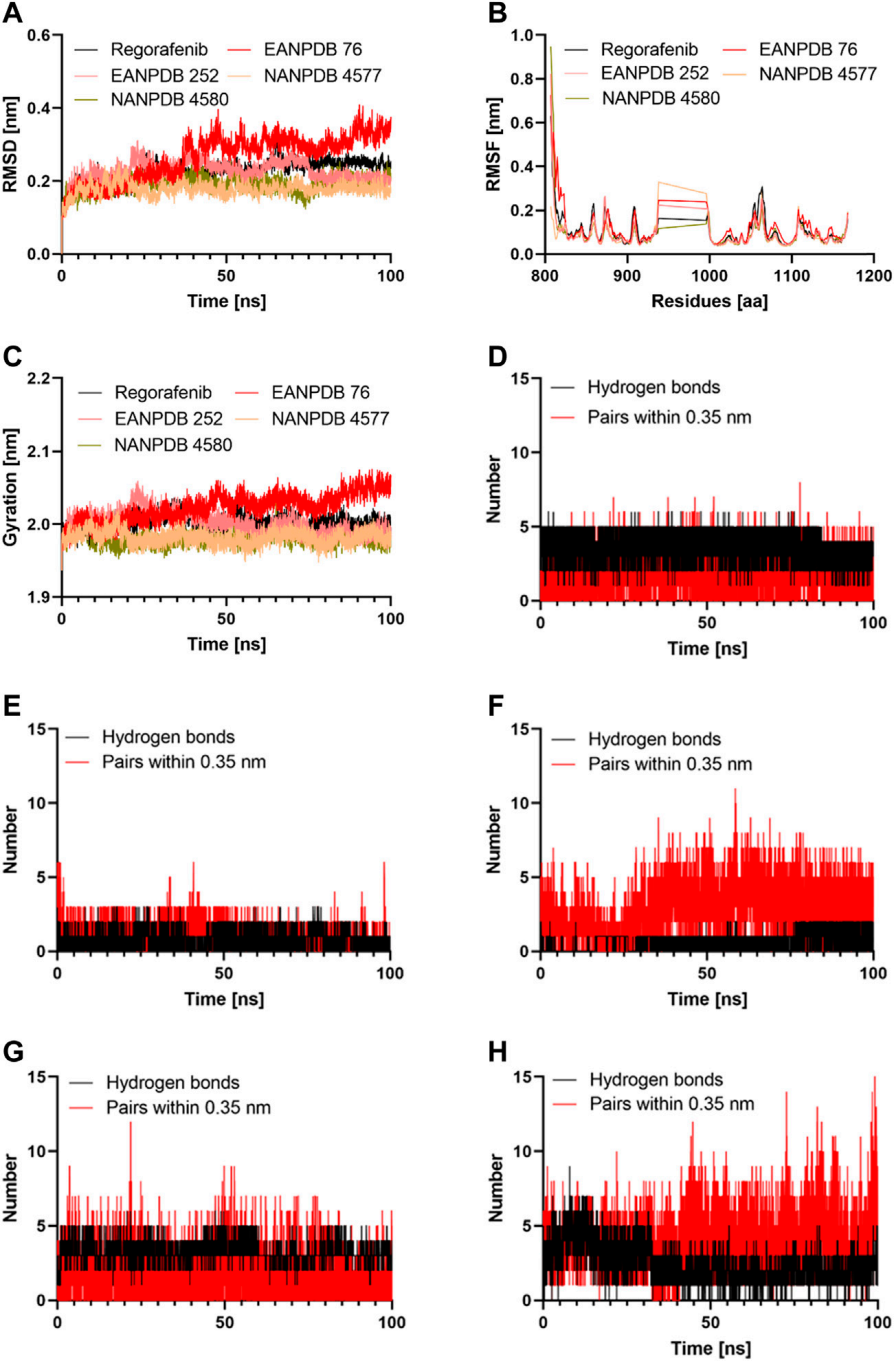
The results of the molecular dynamics study: **(A)** Time evolution of the backbone of the selected complex; **(B)** The comparative RMSF values for the selected compounds; **(C)** The comparative Radius of gyration values for the selected compounds; **(D–H)** The comparative hydrogen bonds and pairs within 0.35 nm for the target protein with the Regorafenib, EANPDB 76, EANPDB 252, NANPDB 4577, NANPDB 4580 respectively.

The virtual and ADMET screening methods were used to choose the top four compounds from a library containing 13336 molecules. Further, molecular dynamics (MD) simulations were conducted for a duration of 100 ns to better understand the binding mechanism and dynamic behavior of these compounds in complex with VEGFR-2 (Figure 3).

### Stability of the docked complexes

Molecular dynamics simulation is a method that could provide a real-time assessment of receptor-ligand interactions. Unlike molecular dynamics simulation, which can show how ligand binding evolves over time in terms of conformational changes, molecular docking can only represent a single position of the ligand-protein complex (Almehmadi et al., 2022). Therefore, we simulated the complexes of VEGFR-2-Regorafenib, EANPDB 76, EANPDB 252, NANPDB 4577, and NANPDB 4580 for 100 ns using molecular dynamics to learn more about the binding interactions between these molecules. These compounds were identified as the top molecules based on the docking results. In the first 30 ns, the four possible VEGFR-2 inhibitors (EANPDB 76, EANPDB 252, NANPDB 4577, and NANPDB 4580) and the reference ligand (Regorafenib) exhibited backbone RMSD values between 0.1 and 0.4 nm (Figure 3A), suggesting that these molecules would adapt to a new conformation within the binding pocket (Mahmood et al., 2022). Subsequently, all systems except EANPDB 76 clearly reached the plateau after 30 ns, having shown a deflection of 0.19 nm and 0.15 nm at 65 ns in the case of EANPDB 252 and NANDB 4580, respectively. Compared with the Regorafenib-VEGFR-2 system, the complexes EANPDB 252, NANPDB 4577, and NANPDB 4580-VEGFR-2 demonstrated better stability. This suggests that these three derivatives may have a higher binding strength with VEGFR-2 for VEGFR-2 than Regorafenib.

### Root mean square fluctuation (RMSF) analysis

The root mean square fluctuation (RMSF) of the molecular dynamics trajectory of a protein is used to characterize changes in residue flexibility (Tabti et al., 2022). Figure 3B shows that the RMSF profiles of the four systems were very similar. Consistent with previous findings, the Lys941-Met1016 residues were the most flexible, suggesting that they play a minor role in receptor-ligand interactions. Among these residues, the region containing Thr940-Glu989 residues were reported not to significantly affect VEGFR-2 catalytic activity (McTigue et al., 1999). Moreover, the Stability around Ala866-Lys941 and Met1016-Arg1050 suggested that these residues were important for binding of the potential inhibitors to VEGFR-2. Both areas represented stable hydrogen interaction zones (Zhang et al., 2013). They contained important residues such as Cys917, Cys919, and Asp1046. These residues formed hydrogen bonds with the inhibitors and reduced the flexibility of the corresponding domains. In this study, we discovered that the three natural derivatives formed hydrogen bonds with Asp1046, Cys919, or Lys868, leading to identical RMSF profiles. Indeed, our findings showed that EANPDB 252, NANPDB 4577, and NANPDB 4580 were more stable than the Regorafenib-VEGFR-2 complex, whereas the EANPDB 76-VEGFR-2 complex was more flexible.

### Radius of gyration analysis

We examined how the compactness of the protein’s structure is altered by binding to different compounds. For this purpose, we calculated the radius of gyration (Rg) as a function of time (Figure 3C). Consisten with the results of RMSD analysis of the protein backbone, EANPDB 252, NANPDB 4577, and NANPDB 4580 exhibited a higher degree of protein compactness than Regorafenib. In addition, the systems containing FDA-approved inhibitors (Regorafenib) showed a relatively stable protein compactness profile. The VEGFR-2-NANPDB 4577 system, which was the most compact of all systems, had an average value of 1.98 nm, whereas the VEGFR-2- NANPDB 4580, VEGFR-2 - EANPDB 252 complexes had an average value of 2.00 nm. The VEGFR-2-ANDB-76 system had an exceptional profile (2.02 nm).

### Hydrogen bonds analysis

Both the affinity for the ligand and its stability depends on the H-bonds that take place between the protein and the ligand, and it increases in direct proportion to the number of H-bonds present in the system (Chen et al., 2016). The Gromacs gmx_hbond module was used to estimate the number of H-bonds formed between the protein and its ligands during 100 ns. The results of this estimation are shown in Figures 3D–G. Regorafenib was found to form an average of 2.01 hydrogen bonds and 3.23 bond pairs within 0.35 nm of the active pocket of VEGFR-2. Similarly, NANPDB 4577 was linked to VEGFR-2 in the binding site via an average of 3.97 hydrogen bonds, while the average number of pairs within 0.35 nm was 4.15, followed by VEGFR-2 and NANPDB 4580, VEGFR-2 and NANPDB 252 with an average number of hydrogen bonds of 1.5 and 1, and an average number of pairs within 0.35 nm of 2.1 and 3.2, respectively. However, for VEGFR-2/NANPDB 4580, the average number of hydrogen bonds was 2.48 and the average number of pairs within 0.35 nm was 3.45. Although the latter two compounds formed fewer hydrogen bonds compared to the reference compound (Regorafenib), they still created stable interactions in the active site of VEGFR-2 by forming hydrogen bonds with crucial residues. The four derivative compounds (EANPDB 252, NANPDB 4577, and NANPDB 4580) were found to be equivalent to Regorafenib and were able to effectively target VEGFR-2 against angiogenesis, as shown by the results of molecular dynamics simulations, despite minor differences between these inhibitors.

### MM-PBSA binding free energy analysis

The binding free energy calculations were performed using the Python script MmPbSaStat.py from the package g_mmpbsa to calculate the average binding free energy for the entire trajectory and to further investigate the interactions of the selected complexes (Jayaraman et al., 2021) (Table 3). Using the output files from g_mmpbsa, this script determines the average free energy of binding and the associated standard deviation/error.

**TABLE 3:** MMPBSA calculations of the predicted binding free energy for all complexes.

Complex	Binding energy (kJ/mol)	SASA energy (kJ/mol)	Polar solvation energy (kJ/mol)	Electrostatic energy (kJ/mol)	Van der Waals energy (kJ/mol)
Regorafenib	−148.036 ± 15.297	−24.050 ± 0.886	197.641 ± 14.272	−78.883 ± 11.262	−242.744 ± 12.705
NANPDB 4577	−161.152 ± 17.683	−32.087 ± 1.327	185.220 ± 15.749	−25.337 ± 9.798	−288.948 ± 14.543
NANPDB 4580	−109.961 ± 17.902	−30.095 ± 1.274	277.253 ± 32.965	−70.428 ± 22.50	−286.691 ± 14.557
EANDB 252	−119.993 ± 24.429	−24.234 ± 1.702	165.665 ± 18.515	−36.976 ± 14.606	−225.447 ± 25.470
EANPDB 76	−103.915 ± 15.884	−29.636 ± 1.075	296.150 ± 18.383	−116.413 ± 13.559	−254.017 ± 15.477

Binding energy indicates how much energy is released when a bond is formed or when a ligand and a protein interact. Several types of energy were considered when calculating binding energy, namely Van der Waal energy, Electrostatic Energy, Solvent Accessible Surface Energy (SASA), and the Polar solvation energy, which was the only one excluded from the analysis (Bhardwaj et al., 2020). All other energy types had a positive effect on the interaction between VEGFR-2 and various molecules. Of all the identified compounds, NANPDB 4577 showed the lowest binding free energy (−161.152 kJ/mol). This value represents the energy required to bind two molecules together. Regorafenib, a newly developed drug, had the second lowest binding free energy (−148.036 kJ/mol). The connections between VEGFR-2 and three putative anti-angiogenic compounds were found to be mediated mainly by Van der Waals interactions (Evdw) rather than electrostatic interactions (Eele). All three derivatives contributed as strongly to Van der Waals forces as regorafenib, but their electrostatic contributions were much weaker in the case of NANPDB 4577 and EANDB 252. It is important to mention that the MMPBSA energetics has two major issues related to the intrinsic dielectric constant and the determination of the entropic term efficiently and accurately. Based on this, other methods such as experimental assays can be conducted in order to validate the accuracy and reliability of the computational predictions.

## Conclusion

In the present study, a virtual screening method was used to search for potential anticancer drugs against VEGFR-2 among 13313 natural products in the African database. Using molecular docking calculations, and ADMET properties, 4 compounds were identified that, when bound to VEFGR-2, had a binding affinity −11.0 kcal/mol and favorable physiochemical and pharmacokinetic properties. These 4 compounds were then subjected to 100 ns long MD simulations to determine their stability and their binding energy using the MM-PBSA method. Compared to Regorafenib as an approved drug, root means square deviations, root means square fluctuation, radius of gyration, hydrogen bonds, and binding-free energy analysis showed that, with the exception of EANPDB 76, all candidatess, including EANPDB 252, NANPDB 4577, and NANPDB 4580, which are derived from natural sources, can effectively target VEGFR-2, similar to Regorafenib. Therefore, we suggest three substances that can be used in anti-angiogenesis treatment. These compounds are expected to deactivate VEGFR-2 and thus reduce the process of angiogenesis. However, it should be noted that their suitability for clinical use and safety need to be further explored, because *in vitro* and *in vivo* experiments were not performed in our study.

## Data Availability

The original contributions presented in the study are included in the article/supplementary material, further inquiries can be directed to the corresponding authors.
